# Endotoxin, Ergosterol, Fungal DNA and Allergens in Dust from Schools in Johor Bahru, Malaysia- Associations with Asthma and Respiratory Infections in Pupils

**DOI:** 10.1371/journal.pone.0088303

**Published:** 2014-02-11

**Authors:** Dan Norbäck, Pawel Markowicz, Gui-Hong Cai, Zailina Hashim, Faridah Ali, Yi-Wu Zheng, Xu-Xin Lai, Michael Dho Spangfort, Lennart Larsson, Jamal Hisham Hashim

**Affiliations:** 1 Uppsala University, Department of Medical Science, Occupational and Environmental Medicine, University Hospital, Uppsala, Sweden; 2 Division of Medical Microbiology, Department of Laboratory Medicine, University of Lund, Lund, Sweden; 3 Department of Environmental and Occupational Health, Faculty of Medicine and Health Sciences, Universiti Putra Malaysia, UPM, Serdang, Selangor, Malaysia; 4 Primary Care Unit, Johor State Health Department, Johor Bahru, Malaysia; 5 Asia Pacific Research, ALK-Abello A/S, Guanzhou, China; 6 United Nations University-International Institute for Global Health, Kuala Lumpur, Malaysia; 7 Department of Community Health, National University of Malaysia, Kuala Lumpur, Malaysia; Louisiana State University, United States of America

## Abstract

There are few studies on associations between respiratory health and allergens, fungal and bacterial compounds in schools in tropical countries. The aim was to study associations between respiratory symptoms in pupils and ethnicity, chemical microbial markers, allergens and fungal DNA in settled dust in schools in Malaysia. Totally 462 pupils (96%) from 8 randomly selected secondary schools in Johor Bahru, Malaysia, participated. Dust was vacuumed from 32 classrooms and analysed for levels of different types of endotoxin as 3-hydroxy fatty acids (3-OH), muramic acid, ergosterol, allergens and five fungal DNA sequences. Multiple logistic regression was applied. Totally 13.1% pupils reported doctor’s diagnosed asthma, 10.3% wheeze and 21.1% pollen or pet allergy. Indian and Chinese children had less atopy and asthma than Malay. Carbon dioxide levels were low (380–690 ppm). No cat (Fel d1), dog (Can f 1) or horse allergens (Ecu cx) were detected. The levels of *Bloomia tropicalis* (Blo t), house dust mite allergens (Der p 1, Der f 1, Der m 1) and cockroach allergens (Per a 1 and Bla g 1) were low. There were positive associations between levels of *Aspergillus versicolor* DNA and daytime breathlessness, between C14 3-OH and respiratory infections and between ergosterol and doctors diagnosed asthma. There were negative (protective) associations between levels of C10 3-OH and wheeze, between C16 3-OH and day time and night time breathlessness, between cockroach allergens and doctors diagnosed asthma. Moreover there were negative associations between amount of fine dust, total endotoxin (LPS) and respiratory infections. In conclusion, endotoxin at school seems to be mainly protective for respiratory illness but different types of endotoxin could have different effects. Fungal contamination measured as ergosterol and *Aspergillus versicolor* DNA can be risk factors for respiratory illness. The ethnical differences for atopy and asthma deserve further attention.

## Introduction

The large ISAAC international study has shown that the increase of asthma is most pronounced in middle-income countries, especially in Asia Pacific [1]. In Malaysia, a tropical country, previous studies from 1990 to 2001 have found 3–10% asthma and 5–8% wheeze prevalence among school children, with a trend towards an increase of asthma in the 1990s [2]–[4]. We found no later studies on asthma or allergy in Malaysian school children. The school environment is an important indoor environment for school children. Schools can be contaminated by a mixture of air pollutants including moulds, bacteria, allergens, particles, volatile organic compounds (VOCs) and formaldehyde [5]–[6]. A recent review concluded that schools might be important sources of allergen exposures, including cat and dog allergens [7]. Most school environment studies are from industrialized countries in temperate climate zones [5]–[7].

Building dampness and indoor mould exposure is a well recognised cause of respiratory and asthmatic symptoms [8] but epidemiological studies on measured mould and bacterial exposure in schools are limited. Two Swedish studies found associations between levels of viable and total moulds in air [9] and volatile organic compound of possible microbial origin (MVOC) [10] with asthmatic symptoms in children. We found only one study on fungal exposure and respiratory health effects in children in schools in a warmer (subtropical) climate, from Taiwan [11].

Chemical analysis of ergosterol (Erg) [12]and beta-1-3 glucan by the limulus method [13] has been used as a marker of total fungal load. One Chinese school study found an association between concentration of ergosterol in school dust and respiratory infections, significant with mutual adjustment for other microbial compounds [14]. Bacteria and bacterial compounds may also influence allergy and respiratory health. Lipopolysaccharide (LPS, endotoxin) and peptidoglycan are the two most studied bacterial cell-wall compounds. LPS is a chemical marker for Gram-negative bacteria. Peptidoglycan is found in all bacteria but in the largest amounts in Gram-positive bacteria [14]. Endotoxin is mostly measured by the biological limulus test [15] but can be measured by chemical methods as well, analysing 3-hydroxy fatty acids (3-OH FA) from LPS. These methods can detect different types of endotoxin from different types of bacteria [16]. Correspondingly, muramic acid (MuA), an amino sugar, is found exclusively in peptidoglycan. Few epidemiological studies on bacterial markers are available from schools. One recent study from the Netherlands measured endotoxin in schools and homes using the Limulus test. The Limulus test is a biological test system using an extract from the horseshoe crab, *Limulus polyphemus* to detect endotoxin. They found that school exposure to endotoxin was positively associated with non-atopic asthma while home exposure to endotoxin was inversely associated with asthma [17]. One Chinese school study investigated health associations of MuA and endotoxin with different types of 3-OH FA [14]. MuA was consistently protective for asthmatic symptoms. Shorter lengths of 3-OH FA tended to be negatively associated while longer lengths were positively associated with asthmatic symptoms. This suggests that different types of endotoxin can have different health effects.

One recent systematic review on asthma and allergy in children concluded that there is a need for more health studies measuring mould derived components in dust, including those measured by molecular methods [18]. Detection and quantification of selected indoor fungi is now possible by using quantitative Polymerase Chain Reaction (qPCR or sometimes called real time PCR). This molecular method can give quantitative data on the occurrence of the most common indoor moulds, irrespectively of viability [19], [20]. One multi-center EU school study found an association between some types of fungal DNA in settled dust, as well as concentration of viable moulds in classroom air with wheeze, cough and rhinitis and decreased lung function in elementary school children [21].

In tropical areas, indoor levels of house dust mite and tropical mite allergens and moulds are expected to be high due to the warm and humid climate, while the ventilation flow can be expected to be high from natural ventilation. There are few studies on exposure to mould and bacteria in schools in tropical countries [22]. Moreover, we found only two previous studies on allergen levels in schools in tropical countries. One study from Brazil concluded that schools are important sources of exposure to dust mite and cockroach allergens [23]. Another study from Singapore concluded that *Bloomia tropicalis* (tropical storage mite) should be considered as a major allergenic component in dust in Singapore, and that levels of cockroach allergens were higher in schools than in homes [24].

The first aim of this study was to investigate associations between ethnicity and asthma, allergies and respiratory symptom in junior high school pupils in Johor Bahru, Malaysia. The second aim was to measure levels of different types of endotoxin (LPS), muramic acid, ergosterol, selected allergens and five different sequences of fungal DNA relevant for indoor fungal exposure in vacuumed dust from pupils’ classrooms. The third aim was to study associations between detected components in vacuumed dust from the classrooms and the health variables mentioned above.

## Materials and Methods

### Study Population

A total of 8 junior high schools were randomly selected in Johor Bahru, Malaysia. Four classes of grade two students were randomly selected in each school, and 15 students were randomly selected from each class. The study proposal was approved by the Medical Research and Ethics Committee of the National University of Malaysia and all participants gave informed consent. We obtained written consent from the students after explaining to them the purpose of the study and their role in answering the questionnaires. The records of study respondents’ signatures are kept at the National University of Malaysia. The students brought the questionnaire home to answer it together with their parents or guardians, but we got signatures only from the students. The study was performed in 2007, and the Ethical Committee approved this procedure since the study only included questionnaire data and no examinations or clinical tests. The questionnaire study and collection of dust from the schools had permission from Johor State Health Department, the principal of each school and the head teacher of each class involved in the study.

### Assessment of Health Data

We used a self-administered questionnaire which used in previous school studies [9], [14], [25], [26]. It included questions on doctors diagnosed asthma, allergies and current asthma and respiratory symptoms obtained from the large international ISAAC study [1], the European Respiratory Health Survey (ECRHS) [27] and previous Swedish school studies [25]. One set of questions asked about asthma, including doctor’s diagnosed asthma (cumulative incidence), current asthma medication use, and asthma attacks during the latest 12 months. Another set of questions asked about airway symptoms related to asthma during the latest 12 months, without using the phrase “asthma”. These symptoms included: 1) wheezing or whistling in the chest the last 12 months 2) at least one daytime attack of shortness of breath during exercise or while resting during the last 12 months (2 questions). 3) at least one night-time awakening with attacks of breathlessness or tightness in the chest during the last 12 months [14], [25], [26]. The question on wheeze during the last 12 months is identical in the ECRHS [27] and the ISAAC study [1]. Moreover, the questionnaire contained questions on current smoking, and allergy to cat, dog and pollen, and parental allergy/asthma. Finally, there was one question on respiratory infections during the last 3 months. The questionnaire was distributed to the selected pupils the same week as the technical measurements and answered with help of the parents at home. Then a school-nurse went through the questionnaires during a face-to-face interview with the pupils to clarify any uncertainly in the questions. When answering the questionnaires, the pupils had no information on the data being collected from the classrooms.

### Building Inspection and Indoor Climate

Details on construction, building materials and age and signs of dampness or mould growth were noted. Temperature (°C), Relative Humidity (RH, %) and concentration of CO_2_ (ppm) were measured in the classrooms during normal activities within 50 to 70 min with Q-TrakTM IAQ monitor (TSI Incorporated, St. Paul, Minnesota, USA), by logging average values over one minute. The instruments were regularly calibrated.

### Dust Sampling

Settled dust was collected by a 400 W vacuum cleaner provided with a special dust collector (ALK Abello, Copenhagen, Denmark) equipped with a Millipore filter (pore size 6 µm). The filter is made of cellulose acetate, and according to the manufacturer it retains 74% of particles 0.3–0.5 µm, 81% of particles 0.5–1.0 µm, 95% of particles 1–10 µm and about 100% of larger particles (>10 µm). Vacuum cleaning was performed for 4 minutes per sample, 2 minutes on the floor and 2 minutes on other surfaces (desks, chairs) as in previous school studies [9], [14], [25]. Each classroom was divided into two parts, one near the corridor and the one near the windows. One dust sample was collected from each part. From each filter 100 mg of the dust was used for allergen analysis at our laboratory. The remaining dust was sieved through 0.3-mm mesh screen to obtain the fine dust, weighted, and used for analysis of fungal DNA and chemical microbial markers. The total amount of sieved dust was weighed in each sample and total amount of dust on each filter was calculated, adding the 100 mg used for the allergen analysis. A third filter was collected by repeating the sampling procedure in the whole classroom. This filter was analysed for house dust mite, tropical storage mite, and cockroach allergens at the ALK laboratory in Guangzhou, China. All filters were sealed in plastic bags and stored at –20°C until the dust samples were taken for allergen and fungal DNA analysis. The sieved dust samples were stored in a low temperature freezer (−80°C).

### Analysis of Chemical Microbial Markers and Fungal DNA

The sieved dust was analyzed for 3-hydroxy fatty acids (3-OH FA) of 10–18 carbon chain lengths, muramic acid (MuA), and ergosterol (Erg), by using gas chromatography-tandem mass spectrometry [12], [16]. Totally 1 to 3 mg of dust was used for 3-OH FAs and MuAc analysis whereas 7 to 22 mg was used for Erg analysis, following acid methanolysis and derivatization (3-OH FAs, MuAc) and alkaline hydrolysis and silylation (Erg), respectively. Total LPS concentration was calculated from the sum of the concentration of C10, C12, C14 and C16, divided by a factor of four, as each LPS molecule has four molecules of 3-OH [28]. C18 was excluded from the LPS calculation as a result of poor correlation of C18 by this method and the classical Limulus Amebocyte Lysate test [29]. The concentrations of chemical microbial markers were expressed as per gram dust.

The method for analysis of fungal DNA has been previously described [30], [31]. Briefly fungal DNA was extracted from 10 mg of sieved dust and five multiplex reactions were performed in five separate tubes targeting the DNA of the following species: total fungi, *Aspergillus* spp and *Penicillium spp* (*Asp/Pen*), *Aspergillus versicolor (A. versicolor)*, *Stachybotrys chartarum* (*S. Chartarum)* and *Streptomyce*s spp. The reaction targeting *A. versicolor* simultaneously amplified an internal positive control that was used to detect PCR inhibition. The oligonucleotides used for amplification and detection were designed using the design software Primer Express 2.0 (Applied Biosystems, Foster City, CA USA). Amplification and detection was performed on a 7300 Real-time PCR Instrument (Applied Biosystems, Foster City, CA USA) using the Taqman® Universal Master Mix (Applied Biosystems, Foster City, CA USA). The fungi DNA level was expressed as cell equivalents (CE), assuming one sequence per cell [32]. The final result was presented as CE/g dust.

### Allergens Analysis

(Enzyme-Linked Immunosorbent Assay (ELISA) was applied at our laboratory to determine the allergen levels of cat (Fel d 1), dog (Can f 1) (Indoor Biotechnologies Ltd, Manchester, UK), and horse (Equ cx) (Mabtech, Stockholm, Sweden) in the dust using monoclonal antibodies as previously described [25]. The following procedure was used to analyse mite and cockroach allergens. Analysis of samples of settled dust (100 mg) was extracted in 1.5 ml of 0.125 M amoniumhydrogencarbonate buffer by rotating mixing for two hours at room temperature. Samples were then filtered by 0.22 µm syringe filters and stored in 1.5 ml eppendorf tubes at −18°C until analysed for the content of allergen. Allergen levels were determined using two-site sandwich ELISA for house dust mite (Der p 1, Der f 1 and Der m 1), storage mite (Blo t) and cockroach (Bla g 1 and Per a 1) (reagents provided by ALK-Abello A/S, Denmark), using monoclonal antibodies except Blo t ELISA which is used purified species specific polyclonal antibody. The assays were basically performed according to the protocols provided by the suppliers. The dust samples were diluted in phosphate buffered saline containing 0.05% Tween and 0.5% bovine serum albumin (BSA) in serial dilution starting with 1/5 and assayed in duplicates. Allergens (Der p 1, Der f 1 and Der m 1) concentrations were expressed as ng/g dust, allergens of Blo t, Bla g 1 and Per a 1 concentrations were expressed as AU/g dust.

### Statistical Methods

Associations between risk factors and symptoms were examined by multi-level multiple logistic regression, controlling for sex, race, smoking and parental asthma/allergy. Two levels were used, individual level and classroom level. Some models did not work when using a 3-level model (individual, classroom, school), but since similar results were obtained in 3-levels and 2-level models when both types of models could be used, we choose to report data for 2-levels models only. Initially we analysed one 2-level model including gender, race, smoking and parental asthma/allergy. As the next step we analysed associations between concentration of microbial components, allergens and fungal DNA in vacuumed dust and symptoms, adjusting for gender, sex, smoking and parental asthma/allergy. Finally, we analysed associations between risk factors and symptoms by stepwise multiple logistic regression (forward Wald). Significant factors in the stepwise regression models were then entered in 2-level logistic regression models. Odds ratio (OR) with 95% confidence interval (95% CI) was calculated for the logistic regression analyses. Factor analysis was performed for the exposure variable by principal component analysis using varimax rotation. Statistics were performed with the Statistical Package for the Social Sciences (SPSS) 17.0 or the STATA statistical package (for multi-level logistic regression), using two-tailed tests at a 5% significance level.

## Results

The participation rate was 96% (462 of 480 invited pupils, 228 females and 224 males). In total, 52% of responders were girls, with a mean age of 14 years (range 14–16 years). Among the respondents, 43% were Malay, 42% were Chinese and 15% were Indians. The prevalence of smoking was 1.3% in girls and 8.6% in boys. Totally 12.5% reported pollen allergy, 10.2% cat allergy and 8.12% dog allergy. Pet keeping was not very common, whereby 14% had a cat at home and 13% had a dog. The prevalence of doctor’s diagnosed asthma and daytime attacks of breathlessness was high (Table 1). Daytime attacks of breathlessness occurring after exercise was common (36%), attacks at rest were less common (10%). Totally 3.5% had current asthma medication and 22.0% had a father or mother with asthma or allergic rhinitis (parental asthma/allergy). The prevalence of doctor’s diagnosed asthma differed between the three ethnic groups (9.3% asthma in Chinese, 4.3% in Indian and 19.9% in Malay pupils). Moreover, reported pollen or pet allergy (atopy) differed between the groups (11.9% in Chinese, 27.5% in Indians and 28.2% in Malay).

**Table 1:** Prevalence of asthma and respiratory symptoms among pupils (N = 462) from junior high schools in Johor Bahru, Malaysia.

Symptoms	Overall (%)	Male (%)	Female (%)	*P*-value	Malay (%)	Chinese (%)	Indian (%)	*P*-value
**Doctor’s diagnosed asthma**	13.1	15.6	10.6	0.11	19.9	9.3	4.3	0.001
**Wheeze or whistling in** **the chest**	10.3	9.5	11.0	0.58	14.8	8.9	1.4	0.005
**Any daytime breathlessness** **(rest or exercise)**	40.5	32.4	48.1	0.001	43.6	41.5	29.4	0.12
**Nocturnal attacks of shortness** **of breath**	7.0	5.4	8.5	0.19	10.7	5.2	1.5	0.02
**Airway infections in the last** **3 months**	18.8	12.1	25.2	<0.001	23.4	15.9	27.5	0.10
**Pollen or fury pet** **allergy (atopy)**	21.1	13.5	28.4	<0.001	28.2	11.9	14.5	<0.001

Data on total prevalence and gender differences obtained from ref. 30.

The mean age of the school buildings was 16 years (range of 3–40 years). The schools were 2 to 4 storeys concrete buildings with painted indoor surfaces and the floor surface consisted of concrete without any paint or floor covering. There was no carpet, pot plant or book shelves in any classroom. None of the schools had a mechanical ventilation system or an air conditioning unit in the classrooms. All classrooms had electric fans on the ceiling and were equipped with glass window panes on both sides that were kept open during lectures. Smoking was not allowed in the schools. The mean room temperature was 29°C (range of 27–31°C), which was similar as the mean outdoor temperature (29°C). The mean indoor relative air humidity was 70% (range of 60–78%), similar as the mean outdoor air humidity (73%). The mean CO_2_ concentration in the classrooms was 490 ppm (range of 380–690 ppm), and 410 ppm outdoors. There was on average 45 pupils per classroom (range of 24–42 pupils). Five (16%) classrooms had signs of water leakage on the ceiling but none had visible indoor mould growth.

All classroom contained detectable levels of 3-OH from endotoxin, MuA and ergosterol. Longer chain C14, C16 and C18 3-OH were most common while C10 and C12 3-OH were found in lower concentrations (Table 2). We found total fungal DNA and *Asp/Pen* DNA in all dust samples. *A. versicolor* DNA, *S. chartarum* DNA, and *Streptomyces* DNA were detected in 87%, 31%, and 31% of the classrooms, respectively (Table 3). *S. chartarum* DNA was common but the levels were very low (range <1–5 CE/g dust). We found low levels of cat allergen (Fel d 1) in three samples (130, 150 and 330 ng/g dust, respectively), but no dog allergen (Can f 1) or horse allergen (Equ cx) in any sample. House dust mite (HDM) allergens were present in low levels in all classrooms, the highest levels were found for Der m 1. The maximum concentration was 30 ng/g dust for Der p 1, 50 ng/g for Der f 1 and 230 ng/g for Der m 1. Low levels of tropical storage mite allergens (Blo t) were found in three samples (0.6, 1.2 and 5.3 AU/g, respectively). American cockroach allergens (Per a 1) or German cockroach allergens (Bla g 1) was found in 59% of the classrooms, the maximum level was 270 U/g Per a 1 and 60 U/g Bla g 1 (Table 3).

**Table 2:** Concentration of chemical microbial markers in settled dust and amount of fine dust in the filters from classrooms (N = 32) in junior high schools in Johor Bahru, Malaysia.

Type of markers	AM	GM (GSD)	Min-Max value
**C10 3-OH FA (nmol/g)**	3.55	3.35 (1.43)	1.20–5.70
**C12 3-OH FA (nmol/g)**	13.38	12.86 (1.33)	7.80–23.60
**C14 3-OH FA (nmol/g)**	38.63	36.44 (1.43)	17.10–70.30
**C16 3-OH FA (nmol/g)**	67.35	65.52 (1.27)	17.10–100.20
**C18 3-OH FA (nmol/g)**	44.42	42.84 (1.43)	21.80–67.90
**LPS (nmol/g)**	41.83	40.62 (1.29)	21.30–60.10
**MuA (µg/g)**	25.69	23.39 (1.56)	9.10–61.40
**Ergosterol (µg/g)**	3.18	2.66 (1.78)	1.10–13.09
**Fine dust (mg)**	1157	1035 (1.66)	214–2412

The concentrations of all studied chemical microbial markers were above the detection limit in all classrooms.

AM = arithmetic mean, GM = Geometric Mean, GSD = Geometric standard deviation.

3-OH FA: 3-hydroxy fatty acids; LPS: Lipopolysaccharide (endotoxin); MuA: Muramic Acid.

**Table 3:** Concentration of fungal DNA and allergens in settled dust from classrooms (N = 32) in junior high schools in Johor Bahru, Malaysia.

Fungal DNA/Allergens	AM	GM (GSD)	Min-Max value	Classrooms with levels above the detection limit (%)
**Fungal DNA(CE/g dust)**				
Total fungal DNA	3.04*10^5^	1.76*10^5^ (3.8)	4.47*10^3^–8.19*10^5^	100
*Asp/Pen* DNA	2.09*10^5^	0.97*10^5^ (7.2)	35–6.03*10^5^	100
*A. Versicolor* DNA	102	24 (12.5)	<1–1180	88
*S. Chartarum* DNA	1	NA	<1–5	31
*Streptomyces* DNA	11	6 (10.9)	<1–115	84
**HDM allergens (ng/g dust)**				
Der p 1	11	9 (1.9)	2–33	100
Der f 1	15	12 (1.9)	4–50	100
Der m 1	52	38 (2.2)	10–230	100
Sum of HDM	77	61 (2.0)	16–270	100
**CR allergens (U/g dust)**				
Per a 1	24	NA	<1–270	44
Bla g 1	9	NA	<1–60	50
Per a 1+Bla g 1	33	5 (9.6)	<1–330	59

AM = arithmetic mean, GM = Geometric Mean, GSD = Geometric standard deviation.

*Asp/Pen: Aspergillus/Penicillium, A. versicolor: Aspergillus versicolor, S. Chartarum: Stachybotrys chartarum.*

HDM = house dust mites, CR: Cockroach.

Factor analysis is a statistical method for data reduction, used to identify groups of variables (factors) associated with each other. Factor analysis was applied to study associations between different types of measured exposure. Four factors were identified. The first factor was related to gram-negative bacteria, HDM and amount of dust. It consisted of C12, C14, C16, C18 3-OH FAs, LPS, sum of HDM allergens and amount of fine dust. Fine dust was negatively associated with the other variables, the more fine dust the lower concentration of endotoxin. The second factor was related to some other types of bacteria mould. It included C10 3-OH FA, MuA and ergosterol. The third factor was related to general DNA sequences from mould. It consisted of total fungal DNA and Asp/Pen DNA. The fourth factor was related to three specific mould species. It consisted of specific DNA from *A. versicolor, S. Chartarum* and *Streptomyces spp.* The sum of cockroach allergens did not belong to any factor.

Initially, association between symptoms and demographic data was analysed in a multiple logistic regression model, including gender, race, smoking and parental asthma/allergy. Girls had more daytime attacks of breathlessness, atopy and respiratory infections, and smokers had more wheeze. Totally 9% of the boys but only 1% of the girls were smokers (p<0.001). Students with parental asthma or allergy had more wheeze, atopy and doctor’s diagnosed asthma. Finally, Chinese pupils had less atopy and less doctors’ diagnosed asthma, and Indian pupils had less wheeze and less doctors’ diagnosed asthma, as compared to Malay pupils (Table 4).

**Table 4:** Associations between respiratory health among pupils (N = 462) from junior high schools in Johor Bahru, Malaysia and demographic data.

Type of factors	Wheeze	Daytime attacks of breathlessness	Nocturnal attacks of breathlessness	A history ofatopy	Respiratory infections last3 months	Doctor’s diagnosed asthma
**Female gender**	0.93 (0.46–1.88)	1.64 (1.09–2.49)*	1.02 (0.45–2.30)	2.12 (1.23–3.63)**	2.46 (1.43–4.22)***	0.49 (0.27–0.92)*
**Chinese**	0.80 (0.40–1.63)	1.20 (0.77–1.87)	0.53 (0.23–1.23)	0.38 (0.21–0.68)***	0.69 (0.40–1.20)	0.42 (0.22–0.79)**
**Indian**	0.09 (0.01–0.74)*	0.61 (0.32–1.17)	0.16 (0.02–1.24)	1.19 (0.59–2.38)	0.66 (0.29–1.48)	0.15 (0.04–0.52)**
**Tobacco smoking**	3.81 (1.15–12.7)*	2.06 (0.81–5.26)	0.62 (0.08–5.18)	0.76 (0.23–2.54)	0.93 (0.26–3.42)	2.34 (0.82–6.72)
**Parental asthma/allergy**	2.67 (1.32–5.39)**	1.49 (0.92–2.41)	2.11 (0.92–4.86)	4.28 (2.50–7.33)***	1.38 (0.78–2.44)	2.45 (1.30–4.61)**

Reported data are Odds Ratios (OR) with 95% Confidence Interval (CI), including gender, race, tobacco smoking, and parental asthma/allergy (heredity) in the multiple logistic regression models.

Race was analysed as categorical variable with Malay race as reference category.

*p<0.05:

**p<0.01;

***p<0.001.

Initially we analysed each exposure variable separately in 2-level multiple logistic regression models, controlling for sex, race, smoking and parental asthma/allergy. There were positive associations between C12 3-OH FA (P = 0.03) as well as C14 3-OH FA (P = 0.007) and sum of HDM allergens (P = 0.03) and respiratory infections. There was a tendency of associations between *A. versicolor* DNA and daytime attacks of breathlessness (P = 0.09) and between ergosterol and doctors diagnosed asthma (P = 0.13). There were negative (protective) associations between the concentration of C10 3-OH FA and wheeze (P = 0.004), and between C16 3-OH FA and daytime attacks of breathlessness (P = 0.009) as well as night time attacks of breathlessness (p = 0.02). Moreover there were negative associations between LPS and daytime attacks of breathlessness (P = 0.03) as well as night time attacks of breathlessness (P = 0.04). In addition there were negative associations between amount of fine dust and respiratory infections (p<0.001) and between concentration of *S. Charatrum* DNA (P = 0.04) as well as *Asp/Pen* DNA (P = 0.04) and doctors’ diagnosed asthma (Table 5–6).

**Table 5:** Associations between respiratory health among pupils (N = 462) from junior high schools in Johor Bahru, Malaysia and concentration of chemical microbial markers in vacuumed dust from the classroom.

Type of exposures	Wheeze	Daytime attacks of breathlessness	Nocturnal attacks of breathlessness	A history of atopy	Respiratoryinfectionslast 3 months	Doctor’s diagnosed asthma
**C10 3-OH FA**	**0.54 (0.35–0.82)** **	0.94 (0.71–1.24)	0.80 (0.54–1.17)	0.93 (0.74–1.18)	0.73 (0.38–1.41)	1.20 (0.92–1.58)
**C12 3-OH FA**	0.98 (0.86–1.12)	0.94 (0.87–1.01)	0.91 (0.80–1.03)	0.97 (0.90–1.04)	**1.21 (1.02–1.45)** *	1.01 (0.93–1.09)
**C14 3-OH FA**	0.90 (0.60–1.36)	0.82 (0.65–1.04)	0.78 (0.55–1.11)	1.05 (0.86–1.30)	**2.07 (1.22–3.51)** **	1.17 (0.92–1.48)
**C16 3-OH FA**	0.85 (0.62–1.15)	**0.78 (0.64–0.94)** **	**0.71 (0.53–0.94)** *	0.96 (0.81–1.13)	0.92 (0.56–1.53)	0.89 (0.73–1.08)
**C18 3-OH FA**	0.93 (0.59–1.46)	0.85 (0.65–1.10)	0.78 (0.53–1.14)	1.06 (0.84–1.34)	1.69 (0.90–3.16)	1.00 (0.76–1.32)
**LPS**	0.82 (0.49–1.36)	**0.72 (0.53–0.97)** *	**0.64 (0.41–0.98)** *	1.00 (0.76–1.31)	1.72 (0.82–3.62)	1.00 (0.72–1.38)
**MuA**	0.69 (0.42–1.12)	0.83 (0.63–1.08)	0.77 (0.50–1.18)	1.14 (0.91–1.43)	0.88 (0.45–1.72)	1.07 (0.83–1.38)
**Ergosterol**	0.94 (0.73–1.20)	0.96 (0.84–1.10)	0.90 (0.71–1.14)	0.95 (0.84–1.08)	1.03 (0.76–1.41)	1.10 (0.98–1.23)
**Amount of fine** **dust on filters**	0.70 (0.25–1.95)	1.43 (0.79–2.58)	1.10 (0.45–2.69)	1.30 (0.71–2.39)	**0.12 (0.04–0.41)** ***	1.08 (0.64–1.82)

AM = arithmetic mean, GM = Geometric Mean, GSD = Geometric standard deviation.

3-OH FA: 3-hydroxy fatty acids; LPS: Lipopolysaccharide (endotoxin); MuA: Muramic Acid.

Reported data are Odds Ratio (OR) with 95% Confidence Interval (CI) by a 2-level hierarchic logistic regression model adjusted for gender, race, tobacco smoking and heredity:

(OR calculated for 10 nmol/g dust increase in C10 3-OH FA).

(OR calculated for 10 nmol/g dust increase in C12 3-OH FA).

(OR calculated for 100 nmol/g dust increase in C14 3-OH FA).

(OR calculated for 100 nmol/g dust increase in C16 3-OH FA).

(OR calculated for 100 nmol/g dust increase in C18 3-OH FA).

(OR calculated for 100 nmol/g dust increase in LPS).

(OR calculated for 10 µg/g dust increase in MuA).

(OR calculated for 1 µg/g dust increase in ergosterol).

(OR calculated for 1000 mg increase in fine dust).

*p<0.05:

**p<0.01;

***p<0.001.

**Table 6:** Associations between respiratory health among pupils (N = 462) from junior high schools in Johor Bahru, Malaysia and concentration of fungal DNA and allergens in vacuumed dust.

Type of exposures	Wheeze	Daytime attacks ofbreathless- ness	Nocturnal attacksof breathless-ness	A historyof atopy	Respiratoryinfectionslast 3 months	Doctor’s diagnosed asthma
***Asp/Pen*** ** DNA**	0.96 (0.71–1.30)	0.92 (0.77–1.11)	0.85 (0.65–1.13)	0.89 (0.76–1.04)	0.96 (0.61–1.49)	**0.82 (0.68–0.99)** *
***A. versicolor*** ** DNA**	0.79 (0.52–1.19)	1.12 (0.98–1.29)	0.91 (0.70–1.17)	0.98 (0.88–1.09)	0.57 (0.36–1.26)	0.91 (0.77–1.08)
***Streptomyces*** ** DNA**	0.32 (0.66–1.65)	1.22 (0.50–2.95)	0.99 (0.27–3.66)	0.85 (0.39–1.84)	0.25 (0.03–2.18)	0.97 (0.40–2.36)
***S. Chartarum*** ** DNA**	0.76 (0.49–1.17)	0.84 (0.66–1.06)	0.62 (0.36–1.08)	1.03 (0.85–1.26)	0.96 (0.55–1.71)	**0.73 (0.54–0.99)** *
**Total fungal DNA**	1.01 (0.82–1.24)	0.94 (0.82–1.06)	0.88 (0.73–1.07)	0.96 (0.86–1.07)	0.91 (0.67–1.24)	0.92 (0.81–1.05)
**Sum of HDM allergens**	1.61 (0.66–3.91)	0.75 (0.46–1.35)	0.90 (0.41–2.00)	0.90 (0.57–1.42)	**3.31 (1.12–9.80)** *	0.80 (0.45–1.43)
**Sum of CR allergens**	1.14 (0.56–2.43)	0.81 (0.52–1.26)	1.23 (0.71–2.14)	0.73 (0.48–1.11)	0.42 (0.10–1.73)	0.44 (0.20–1.02)

*Asp/Pen: Aspergillus/Penicillium, A. versicolor: Aspergillus versicolor, S. Chartarum: Stachybotrys chartarum.*

HDM = house dust mites, CR: Cockroach.

Reported data was Odds Ratio (OR) and 95% Confidence Interval (CI) by a 2-level hierarchic logistic regression model adjusted for gender, race, tobacco smoking and heredity:

(OR calculated for 10^5^ CE/g dust increase in total fungal DNA).

(OR calculated for 10^5^ CE/g dust increase in *Asp/Pen* DNA).

(OR calculated for 100 CE/g dust increase in *A. versicolor* DNA).

(OR calculated for 100 CE/g dust increase in *Streptomyces* DNA).

(OR calculated for 1 CE/g dust increase in *S. chartarum* DNA).

(OR calculated for 100 ng/g dust increase in sum of house dust mite (HDM) allergens).

(OR calculated for 100 ng/g dust increase in sum of cockroach (CR) allergens).

*p<0.05:

**p<0.01;

***p<0.001.

As a next step we applied stepwise regression analysis (forward, Wald) to select significant variables in six combined models (one for each health variable). These variables were then entered in six 2-level logistic regression models (pupil and classroom level). Females had more day time breathlessness (p<0.001) and more respiratory infections (p = 0.02) than boys but less doctor’s diagnosed asthma (p = 0.003). Indian children had less wheeze than Malay, Chinese children had less atopy than Malay and both Indian and Chinese children had less doctor’s diagnosed asthma than Malay children (reference group). Concerning the classroom exposure, there was a negative association between the concentration of C10 3-OH FA and wheeze (p = 0.004). There were positive associations between the concentration of *A. versicolor* DNA and daytime breathlessness (P = 0.002) and negative associations between C16 3-OH FA (p = 0.005) and *S. Chartarum* DNA (p = 0.02) and daytime breathlessness. There was a negative association between the concentration of C16 3-OH FA and night time breathlessness (P = 0.04). There was a positive association between C14 3-OH FA and respiratory infections (P = 0.001) and negative associations between LPS (p = 0.001) and amount of fine dust (p = 0.001) and respiratory infections. There was a positive association between the concentration of ergosterol and doctor’s diagnosed asthma (p = 0.04) and a negative association between cockroach allergens and doctor’s diagnosed asthma (p = 0.04) (Table 7).

**Table 7:** Final models (2-level hierachic logistic regression) for associations between respiratory health among pupils (N = 462) from junior high schools in Johor Bahru, Malaysia).

	OR (95% CI)	*P*-value
**Wheeze**		
Chinese	0.92 (0.41–2.07)	0.84
Indian	0.09 (0.01–0.82)	0.03
Smoking	4.71 (1.30–17.06)	0.02
Parental asthma/allergy	2.51 (1.18–5.32)	0.02
C10 3-OH FA	0.54 (0.36–0.82)	0.004
**Daytime attacks of breathlessness**		
Female	2.16 (1.43–3.27)	<0.001
C16 3-OH FA	0.80 (0.68–0.93)	0.005
* A. versicolor* DNA	1.20 (1.07–1.36)	0.002
* S. chartarum* DNA	0.78 (0.64–0.97)	0.02
**Night-time breathlessness**		
C16 3-OH FA	0.87 (0.59–0.98)	0.04
**Pollen or furry pet allergy (Atopy)**		
Chinese	0.36 (0.20–0.63)	0.001
Indian	1.06 (0.54–2.09)	0.86
Parental asthma/allergy	4.19 (2.48–7.07)	<0.001
**Respiratory infection last 3 months**		
Female	2.07 (1.12–3.85)	0.02
C14 3-OH FA	1.18 (1.07–1.30)	0.001
LPS	0.78 (0.68–0.90)	0.001
Amount of fine dust	0.10 (0.03–0.39)	0.001
**Doctor’s diagnosed asthma**		
Female	0.46 (0.25–0.86)	0.003
Chinese	0.35 (0.18–0.65)	0.001
Indian	0.15 (0.04–0.52)	0.003
Parental asthma/allergy	2.65 (1.40–5.02)	0.003
Ergosterol	1.12 (1.01–1.26)	0.04
Cockroach allergens	0.91 (0.83–0.99)	0.04

3-OH FA: 3-hydroxy fatty acids; LPS: Lipopolysaccharide (endotoxin); MuA: Muramic Acid.

*Asp/Pen: Aspergillus/Penicillium, A. versicolor: Aspergillus versicolor, S. Chartarum: Stachybotrys chartarum.*

The models include variables retained in a stepwise logistic regression model (forward regression Wald statistics, p<0.10). These variables were entered in a 2-level hierarchic logistic regression model adjusting for classroom level.

(OR calculated for 10 nmol/g dust increase in C10 3-OH FA).

(OR calculated for 10 nmol/g dust increase in C14 3-OH FA).

(OR calculated for 10 nmol/g dust increase in C16 3-OH FA).

(OR calculated for 10 nmol/g dust increase in LPS).

(OR calculated for 1 µg/g dust increase in ergosterol).

(OR calculated for 1000 mg increase in fine dust).

(OR calculated for 100 CE/g dust increase in *A. versicolor* DNA).

(OR calculated for 1 CE/g dust increase in *S. chartarum* DNA).

(OR calculated for 100 ng/g dust increase in sum of cockroach allergens).

The home is an important indoor environment for students. The questionnaire included questions on the indoor environment at home and a study on associations between the home environment and asthma, respiratory symptoms and allergies will be published separately. Totally 43% reported exposure to any environmental tobacco smoke (ETS) at home and 49% reported any signs of dampness and indoor moulds at home the last 12 months. However, when making further additional adjustments for ETS and dampness/moulds at home in the stepwise regression models (Table 7), all associations between school environment exposure and the medical symptoms remained significant and similar OR were obtained.

## Discussion

Respiratory symptoms, particularly exercise-induced daytime breathlessness and doctor’s diagnosed asthma were common among the school pupils in Johor Bahru, Malaysia. Tobacco smoking was more common in boys than girls and related to wheeze. Moreover, as expected, parental asthma/allergy was a predictor of wheeze, doctor’s diagnosed asthma and atopy. Fungal DNA from *Aspergillus versicolor* was positively associated while fungal DNA from *Stachybotrys sp.* was negatively associated with daytime attacks of breathlessness. Ergosterol, another fungal marker, was positively associated with doctor’s diagnosed asthma. Endotoxin at school seems to be mainly protective for respiratory illness but different types of endotoxin seemed to have different effects C10 3-OH FA and C16 3-OH FA were protective while C14 3-OH FA was a risk factor for respiratory illness.

Particles in indoor environments can be sampled by different methods, sampling different size fractions and different locations in the room. The present study sampled dust by vacuum cleaning of floors and upper horizontal surfaces. The advantage with this method is that it samples dust from all parts of room and collects all size fractions. Moreover, the amount of collected dust is large enough to analyse many types of allergens, chemical microbial compounds and fungal DNA. The disadvantage is that some of the collected particles are large and may not become airborne and thus not inhaled by the students. In the school study from Johor Bahru [30], we have used and compared three different methods to sample particles (vacuuming, Petri dish sampling and swab sampling). The Petri dish method was used to measure airborne furry pet allergens and fungal DNA. Settling particles were sampled by leaving empty Petri dishes (without any agar) in the classroom for one week. Fungal DNA and furry pet allergens were analysed by the same methods as for the vacuumed dust. The advantage with the Petri dish method is that it collects smaller particles that has been airborne. The disadvantage is that it collects particles on a very small surface area which may not be representative for the whole classroom. Moreover, the amount of collected particles is very small and not enough to analyse many allergens or chemical microbial compounds. The swab sampling collected settled particles on a small surface above the blackboard and was used to analyse mycotoxins and fungal DNA. We have previously reported significant associations between fungal DNA levels (*A. versicolor* and *Streptomyces* DNA) and respiratory health in school children in the Johor Bahru school study, analysing data for particles collected by the Petri dish method [30]. We did not find any correlation between the levels of fungal DNA in the classrooms collected by the Petri dish method and the levels in vacuumed dust presented in this article (unpublished data). Thus we consider the two methods to measure different aspects of indoor fungal contamination, possibly because they sample different size fractions and different parts of the classroom.

We used QPCR to quantify five fungal DNA sequences. QPCR measure the number of DNA copies in the sample. However, we have expressed the results as cell equivalents, assuming one copy per cell. This was done in order to present results comparable with previous studies by us and other research groups using the cell equivalent concept. However, the assumption of one copy per cell may not be true in all cases. It is well known that some fungal species may contain multiple copies, leading to an overestimation of the number of cells. In addition, the DNA extraction efficiency may not be 100% for all fungal species, and poor extraction efficiency may lead to an underestimation of the number of cells. These potential problems with the cell equivalent concept have been previously discussed [31], [32].

Schools, classrooms and students were randomly selected from all secondary schools in Johor Bahru, Malaysia, and the response rate was high. All samples were analysed after questionnaire data was completed, and environmental sampling was conducted the same week as the questionnaire study. Moreover, the students did not have any information on the results of the environmental measurements when they answered the questionnaires. Since Johor Bahru has a similar climate all year around, ventilation flow or indoor levels of pollutants would be expected to be constant throughout the year. Thus we conclude that the study was not seriously influenced by selection or information bias. However, the cross-sectional study design limits the possibility to draw conclusions on causal effects. Another limitation of the study is the lack of clinical data and in the future more school environment studies are needed combining questionnaire data with clinical measurements, e.g. allergy testing by skin prick tests and measurement of nitrogen oxide in exhaled air (FeNO). Both these methods can be used by trained medical personnel in field studies, e.g. in schools.

In the multiple regression analysis, girls reported more daytime attacks of breathlessness, atopy and respiratory infections, but fewer doctor’s diagnosed asthma. Gender differences in this study in Johor Bahru schools have been previously discussed [30]. The prevalence of doctor’s diagnosed asthma was relatively high (13.0%) and differed between the three ethnic groups (9.3% asthma in Chinese, 4.3% in Indian and 19.9% in Malay pupils). In addition the level of self-reported atopy differed (11.9% in Chinese, 27.5% in Indian and 28.2% in Malay). We found no previous study on ethnical differences in asthma or allergy in Malaysia, except a study on asthma-related quality of life in asthmatic adults in which Indians reported worse quality of life than Malay or Chinese [33]. However, ethnic differences in asthma have been studied in Singapore. One early study found that the cumulative prevalence of doctor’s diagnosed asthma was significantly higher in Malay (6.0%) and Indians (6.6%) as compared to Chinese (3.0%) [34]. A study on temporal trends and ethnic variations in asthma mortality in Singapore 1976–1995 found marked ethnical differences, with 5.0 times higher asthma mortality rate in Malay, and 2.6 times higher rate in Indians as compared to Chinese [35]. It is unclear to what extent these ethnic differences are related to differences in diet, home environment, early childhood exposure, or genetic polymorphism. One study comparing IL4 receptor genetic polymorphism for two SNPs (Ile50/Ile50 and R576R) fount that among normal population in Singapore (blood donors), the prevalence of R576R was lower in Chinese as compared to Malay. Among asthmatics, the prevalence of R576R was lower in Chinese as compared to both Malay and Indian and the prevalence of Ile50/Ile50 among asthmatics was lower in Chinese than in Malay [36]. These two SNPs are correlated with IgE production and atopic asthma.

The level of CO_2_ was low in the classrooms (range 380–690 ppm) and always below the recommended limit of 1000 ppm [37]. This was due to effective natural ventilation since all classrooms had windows with glass panes on both sides of the classrooms. The levels of allergens in the settled dust were low. Cat, dog or horse allergens were not detected in any classroom. Tropical storage mite (*Bloomia tropicalis*) was detected at low levels only in 3 classrooms. House dust mite and cockroach allergens were commonly found but at low levels, GM was 33 U/g dust for cockroach allergens and 77 ng/g dust for HDM allergens. In the final regression models, we found no associations between allergen levels in the dust and any health variables, except for a negative (protective) association between sum of cockroach allergens an respiratory infections during the last 3 months. In the factor analysis, cockroach allergens were not associated with any other measured exposure. We have no explanation to this protective association which could be due to a chance finding. HDM and cockroach allergens have been detect in other school studies from tropical countries. In a school study from Sao Paulo in Brazil,. GM for floor dust samples was 500 ng/g for sum of HDM allergens (Der p1+Der f 1). GM values for the cockroach allergen Bla g 1 was 3.4 U/g in morning samples and 4.7 U/g in afternoon floor dust samples. Animal allergens (Fel d 1 and Can f 1) were remarkably low in all samples [23]. In a school study from Singapore, GM level was 100 ng/g for Der p1, 200 ng/g for Der f 1, 5900 ng/g for Bla g 1, 100 ng/g for dog allergen (Can f 1), 30 ng/g for cat allergen (Fel d 1), and 632 AU/g for tropical storage mites (Blo t) [24]. In conclusion, our data on allergens in Malaysia schools is in agreement with data from Singapore and Brazil, suggesting relatively low levels of these allergens in schools in tropical countries.

We found a positive association between *A. versicolor* DNA and daytime attacks of breathlessness. The majority of the daytime breathlessness was exercise-induced daytime attacks. This association is in agreement with the previous data analysis from the same study, sampling airborne fungal DNA by the Petri dish method [30]. An association between *A. versicolor* DNA in vacuumed dust from classrooms and asthmatic symptoms among pupils has been reported from a European school environment study [21]. *A. versicolor* is often found in damp buildings and produces the mycotoxin sterigmatocystin [38]. Ergosterol was positively associated with doctor’s diagnosed asthma. In a previous school study in Taiyuan in northern China [14], concentrations of ergosterol in classroom dust were positively associated with increased prevalence of respiratory infections (mutual adjustment model). The prevalence of doctor’s diagnosed asthma was very low in this Chinese study (1.2%) and associations for asthma could not be evaluated. The GM for ergosterol was 0.57 µg/gram dust in Taiyuan schools, five times lower than in our Malaysian schools (GM = 2.66 µg/gram dust). In homes, an association between ergosterol concentration in dust and asthmatic symptoms have been previously reported [39]. Surprisingly we found a negative association between *Stachybotrys* DNA and daytime attacks of breathlessness, and the association was significant even after adjusting for the protective effect of endotoxin (C16 3-OH FA). *S. Chartarum* is well known to produce several very toxic macrocyclic trichotecenes, which have profound toxic effects in animal experimental studies [40]. The negative associations found for this species is in agreement with the previous analysis of associations for airborne *Stachybotrys* DNA by the Petri dish method [30] and is possibly due to associations with some other protective factor not identified in this study.

We found a positive association between C14 3-OH FA and respiratory infections the last 3 month. There were negative (protective) associations between C10 3-OH FA and wheeze, between C16 3-OH FA and daytime as well as night time attacks of breathlessness and a negative association between total endotoxin concentration (LPS) and respiratory infections the last 12 months. We found only two previous studies on health associations for endotoxin in schools and respiratory illness in pupils. On school study from the Netherlands, sampled airborne dust by electrostatic dust collectors (EDC) and measured total endotoxin by the Limulus test. In contrast to our study they found positive associations between levels of endotoxin in schools and non-atopic asthma [17]. One Chinese school study, using the same methodology as in our study, concluded that the total concentration of endotoxin (LPS) was positively associated with daytime attacks of breathlessness, but shorter lengths of LPS, C10, C12 and C14 3-OH FAs were negatively associated with either wheezing or daytime attacks of breathlessness [14]. Thus results from our study are partially in agreement with this study, especially for C10 3-OH FA and total LPS. In addition, we found a negative association between the amount of fine dust on the filters and respiratory infections, significant even when adjusting for endotoxin levels. Factor analysis could identify one factor consisting of C12, C14, C16, C18 3-OH FA, total LPS, sum of HDM allergens and amount of fine dust. This factor relates to endotoxin and house dust mite allergen concentration. Fine dust was negatively associated with the other variables, the more fine dust the lower concentration of endotoxin. The negative association for fine dust and respiratory infections could be due to correlation with some other protective factor not identified in this study. One speculative explanation could be that the fine dust comes mainly from the outdoor environment and higher air exchange in the classroom may lead to more fine dust entering the classroom but also less spread of airway infection between the children. However, this speculation is not supported by our measurement data, since we did not find any significant association between amount of fine dust and CO2 levels in the classroom.

In conclusion, gender, smoking and parental asthma/allergy are associated with asthma, asthmatic symptoms and self-reported atopy in pupils in Malaysia. Children in classrooms with a higher concentration of two specific markers of endotoxin (C12 and C14 3-OH FAs) and house dust mites in dust can have a higher risk for respiratory infections. Moreover children in classrooms with a higher concentration of *A. Versicolor* in dust can have a higher risk for daytime attacks of breathlessness. Ergosterol, a general fungal cell-wall component, can be a risk factor for asthma. Certain types of endotoxin, indicated by C10 and C16 3-OH FAs, can be protective for wheeze and night time attacks of breathlessness, respectively. The total amount of endotoxin in vacuumed school dust seemed to be mainly protective for respiratory illness but different types of endotoxin could have different effects. Finally, the ethnic differences for atopy and asthma deserve further attention and more studies are needed comparing health associations between indoor allergens and microbial components sampled by different sampling methods for particles.
